# Design and analysis of tetra-port MIMO antenna for next generation vehicular communication

**DOI:** 10.1038/s41598-026-48861-0

**Published:** 2026-05-02

**Authors:** Lekha Kannappan, Dhananjeyan Rajendran, Rajesh Kumar Dhandapani, Sandeep Kumar Palaniswamy, Sachin Kumar, Bhawna Goyal, Om Prakash Kumar

**Affiliations:** 1 Department of Electronics and Communication Engineering, SRM Valliammai Engineering College, Kattankulathur, 603203 India; 2 Department of Electronics and Communication Engineering, Vel Tech Rangarajan Dr. Sagunthala R&D Institute of Science and Technology, Chennai, 600062 Tamil Nadu India; 3 Department of Electronics and Communication Engineering, Faculty of Engineering and Technology, SRM Institute of Science and Technology, Kattankulathur, 603203 India; 4 Department of Electronics and Communication Engineering, Galgotias College of Engineering and Technology, Greater Noida, 201310 India; 5 University Centre for Research and Development, Chandigarh University, Gharuan, Mohali, 140413 India; 6 Manipal Institute of Technology, Manipal Academy of Higher Education, Manipal, India

**Keywords:** Defected ground structure (DGS), High isolation, Tetra-port MIMO antenna, Vehicular communication, V2V communication, SDG 9—Industry, Innovation, and Infrastructure, SDG 11—Sustainable Cities and Communities, Engineering, Physics

## Abstract

The demand for advanced automotive applications necessitated the development of 5G/6G multiple-input-multiple-output (MIMO) antennas. This work presents a low-profile antenna resonating at 5.9 GHz, suitable for vehicle-to-vehicle communication applications. The resonance is achieved through the use of a defected ground structure and geometric modifications to the radiator. The single element antenna is converted into a MIMO antenna by employing the elements perpendicular to each other. The unit cell antenna has dimensions of 12 mm × 11 mm, and the MIMO antenna measures 42.44 mm × 43.56 mm. The antenna reflection coefficient has been evaluated and is found to be less than −10 dB across the operating band. The tetra-port MIMO antenna achieves greater than 20 dB isolation without the use of any isolation structures. The proposed antenna shows the gain of 3.8 dBi, and efficiency of 87% at the operating frequency. Diversity parameters are evaluated to better understand the performance of the suggested MIMO antenna. The proposed MIMO antenna exhibits an envelope correlation coefficient below 0.1, a diversity gain above 9.9 dB, a total active reflection coefficient below −10 dB, channel capacity loss below 0.4 bits/s/Hz and MEG ratio is close to unity. When installed in the vehicle, the antenna is unaffected by interference from nearby radiators and the metal body of the car.

## Introduction

The rapid development of automotive wireless communication, together with the high data rates and low latency offered by fifth generation (5G) technology, enables vehicle-to-road interaction and supports autonomous vehicle operation^1,2^. The majority of recent applications, including internet of things (IoT), use 5G microwave frequencies to improve vehicle connectivity^3^. The implementation of 5G in vehicles will allow for better vehicular applications such as vehicle-to-vehicle (V2V), vehicle-to-infrastructure (V2I), vehicle-to-pedestrian (V2P), vehicle-to-everything (V2X), and advanced driving assistance systems (ADAS). Effective vehicular communication systems require ensuring road safety, rapid emergency response, and reduced traffic congestion. 5G technology for vehicular communication enhances bandwidth and data rates while significantly lowering latency. This will improve the communication in the crowded areas and aids safety applications like collision avoidance^4,5^. However, the introduction of sixth generation (6G) cellular communication presents a new and appealing opportunity for V2X applications. In comparison to 5G V2V/V2X systems, 6G V2V and V2X systems offer a wider application range, more complex signal analysis, and a higher density of connected vehicles. It also anticipates an increased need for dependability, low latency, and computational resources^6^. The widespread implementation of connected cars has increased the challenge of heterogeneity, instability, and large-scale data management. This provides the background of combining 6G for V2V and V2X applications^7^.

A vehicle must integrate multiple frequencies to support a wide range of information and entertainment systems. This necessitates a multiple number of antennas for each specific application. To avoid the space requirement, multiple bands must be implemented in a single antenna design. Multiple frequencies can be integrated into the antenna via techniques such as meandering lines, split ring resonator (SRR), reconfigurability, and photonic wideband gap devices^8^. Metamaterials with complex structures, such as SRR and complementary split ring resonators (CSRR), result in complex antenna structures^9^. The reconfigurable antenna operates on multiple bands using diodes or transistor. The number of diodes increases in proportion to the number of frequencies^10^. Meandering line techniques are an effective way to achieve multiple frequencies without increasing complexity or size^11,12^.

With the rapid growth of technology among users, there is a significant need for compact antennas that support higher data rates and wider bandwidth. Multiple-input-multiple-output (MIMO) technology offers a practical approach to addressing these challenges in vehicular communication systems^13^. MIMO can efficiently overcome multipath propagation by allowing data to be transmit and receive over multiple channels at the same time^14,15^. To improve the performance of the MIMO antenna, it is necessary to ensure low correlation between the signals received by it. When antennas are placed close together in compact devices, mutual coupling between them should be minimized to ensure optimal system performance. The increase in mutual coupling and correlation reduces channel capacity and diversity gain (DG)^16,17^. To overcome mutual coupling, techniques such as neutralization lines, parasitic elements, slot elements, decoupling networks, and increasing the space between antennas are required^18,19^. MIMO performance can be assessed using diversity parameters such as envelope correlation coefficient (ECC), DG, total active reflection coefficient (TARC), and channel capacity loss (CCL)^20,21^. In^22^, a 5G MIMO antenna is designed to operate in the 28/38 GHz frequency bands. Meandering lines are used to achieve dual-band resonance. The MIMO antenna has an ECC of less than 0.0001 and a DG of more than 9.99 dB, making it useful for millimetre-wave cellular communication. In^23^, a four-port MIMO antenna that resonates in the 26–40 GHz band is presented. Three circular rings are used to achieve an operating frequency with ECC < 0.001, DG > 9.9 dB, and TARC < −10 dB. In^24^, a flexible four-port MIMO antenna is reported for 5G applications operating at frequencies of 27.76–28.15 GHz, 32.02–32.46 GHz, and 37.39–38.586 GHz. The MIMO antenna has ECC < 0.001, DG of 10 dB, CCL of 0.15 bits/sec/Hz, and TARC of less than −10 dB. A 5G mm wave network antenna is designed in^25^ and operates in the 24.4 to 27.9 GHz frequency range. The rectangular patch contains two semi-circular stubs to increase bandwidth, and the antenna has an ECC of less than 0.001 with its DG exceeds 9.4 dB. Vehicular communication requires antennas that can operate in complex and dynamic electromagnetic environments^26^. The metallic body of the vehicle can cause impedance mismatch, distortion in radiation pattern and efficiency due to electromagnetic coupling^27^. Furthermore, the vehicular environment suffers severe multipath propagation due to buildings, nearby vehicles, and roadside structures, causing signal fading^28^. Polarization diversity and pattern diversity techniques are used to improve signal reception in various propagation scenarios. Additional challenges arise when integrating antennas into vehicles, such as limited space, aesthetic requirements, and aerodynamic constraints. Designing compact vehicular antennas with high performance under these practical constraints remains a critical research challenge^29^.

Microstrip antennas have a wide range of applications in the sub-6 GHz spectrum due to their low-profile and ease of fabrication. The 5.9 GHz frequency is a 5G mid-band within the sub-6 GHz band. Sub-6 GHz frequencies are critical for 6G because they provide deep penetration, wide area coverage, and MIMO/beamforming technology^30,31^. In this paper, a four-port MIMO antenna is designed with isolation greater than 20 dB without the use of additional structures such as metasurfaces or parasitic elements. The antenna resonates at 5.9 GHz, with a reflection coefficient of less than −10 dB. The antenna resonates in the intelligent transportation systems (ITS)/V2X band, making it appropriate for vehicle communication systems. The antenna facilitates dedicated short-range communications (DSRC), V2V, and V2I communication. The antenna is investigated for the required scattering and diversity parameters, demonstrating that it is reliable in a vehicular environment.

## Antenna design

### Development of a single element

**Fig. 1:**
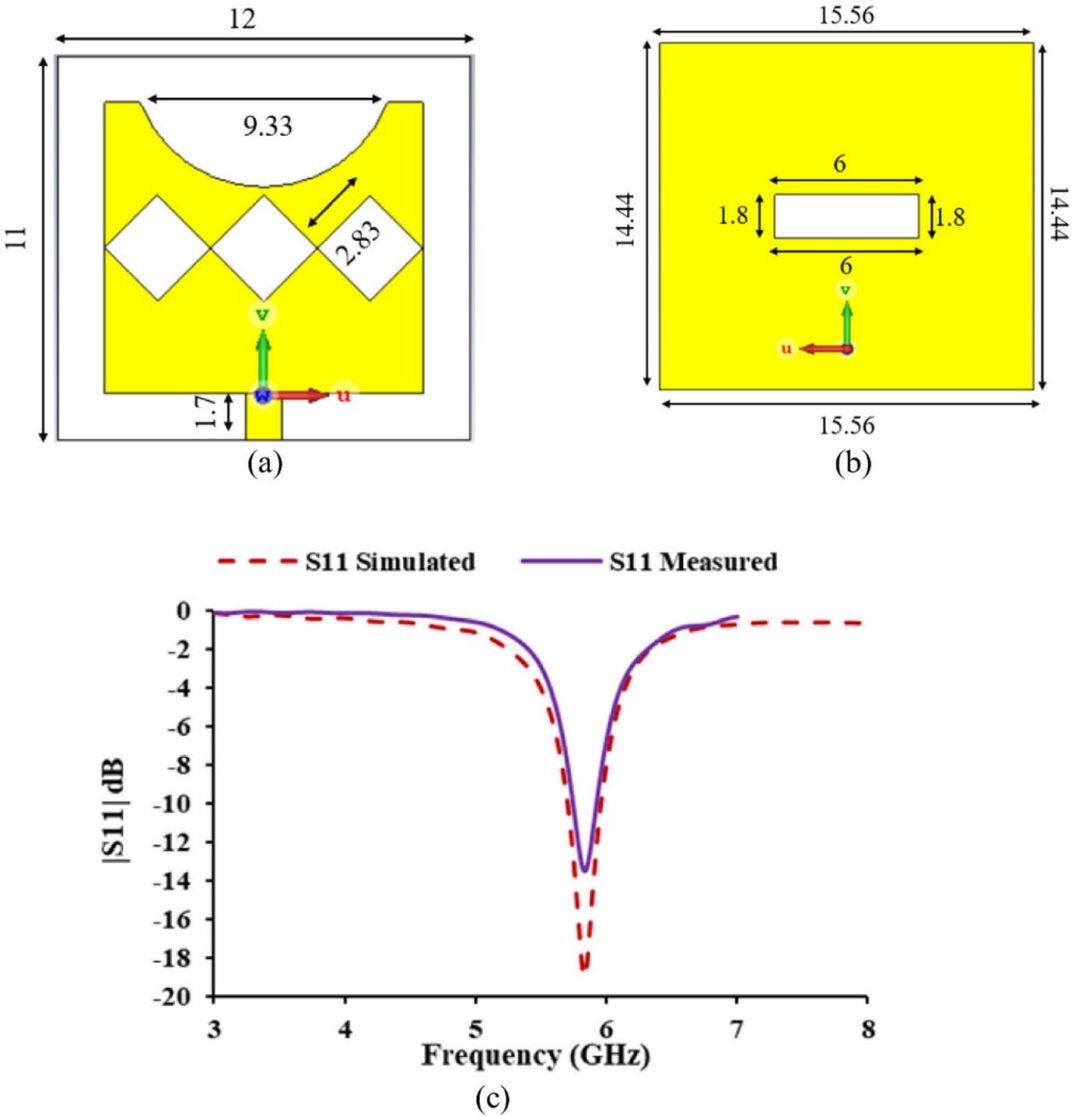
Designed unit cell antenna (dimensions in mm): (**a**) Front view, (**b**) Back view, (**c**) Simulated and measured reflection coefficients.

Figure 1a depicts the design of the unit cell antenna, while Fig. 1b shows its reflection coefficients. The unit cell antenna is developed on a 1.6 mm thick FR-4 substrate. The FR-4 substrate is chosen due to its low cost and widespread availability. The antenna is designed with a basic rectangular patch and a full ground plane. A semicircular slot and three rhombus-shaped slots are incorporated in the radiator to achieve resonance at the desired operating frequency. A rectangular slot is loaded in the ground surface of the unit cell to improve impedance matching. The unit cell antenna and its MIMO configuration are simulated with CST Microwave Studio.

**Fig. 2:**
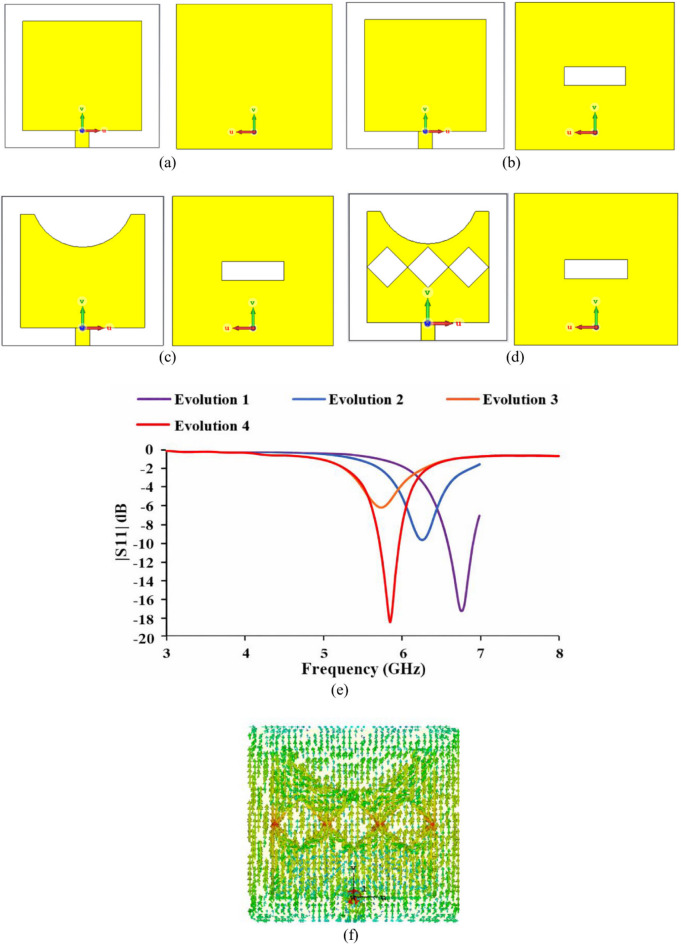
Antenna evolution: (**a**) Evolution 1, (**b**) Evolution 2, (**c**) Evolution 3, (**d**) Evolution 4, (**e**) Reflection coefficients of the evolution stages, (**f**) Surface current distribution at 5.9 GHz.

Figure 2 depicts the evolution of the unit cell antenna. The unit cell antenna has a simple rectangular patch and a complete ground plane, as shown in Fig. 2a. A full ground plane provides a stable and continuous reference for current distribution, which improves radiation efficiency and impedance matching. Also, a full ground plane helps in achieving a more uniform radiation pattern and reduces unwanted back radiation. Furthermore, a full ground offers better shielding of the feed and reduce interference from nearby electronic components, making the unit cell suitable for multi-functional vehicular communication systems. In Fig. 2b, a rectangular slot is loaded in the ground surface of the unit cell. The slot acts as a perturbation element that changes the effective electrical length of the unit cell, thereby enabling resonance at the desired operating frequency without changing the size. Furthermore, the rectangular slot could help reduce mutual coupling in MIMO configurations by disrupting surface current paths, thereby improving isolation between antenna elements. Figure 2c shows the measured and simulated reflection coefficients.

In Fig. 2c, a semicircular truncation is introduced in the rectangular patch of the unit cell antenna, which changes the surface current flow on the patch. Due to this modification, the current travels along a longer path, which increases the effective electrical length of the antenna. Consequently, the antenna is able to resonate at the required operating frequency without increasing its physical size. Also, the semicircular cut also helps improve impedance matching by redistributing the current near the radiating edges of the patch. In Fig. 2d, three rhombus-shaped slots are loaded into the patch to control the surface current distribution more effectively. These slots extend the electrical path length of the current, allowing the antenna to resonate at the desired operating frequency (5.9 GHz) without increasing its physical size. Thus, antenna miniaturization is realized. Figure 2e depicts the reflection coefficients of the evolution stages.

Figure 2f shows the surface current distribution of the unit cell antenna at 5.9 GHz. The current is mainly concentrated along the edges of the patch and around the slots, indicating that these regions play a dominant role in radiation. The strong current flow near the slots confirms that the introduced geometrical modifications effectively control the current path. The reduced current concentration in the central region suggests lower coupling between adjacent antenna elements, which could be beneficial for improving isolation in MIMO antenna.

**Fig. 3:**
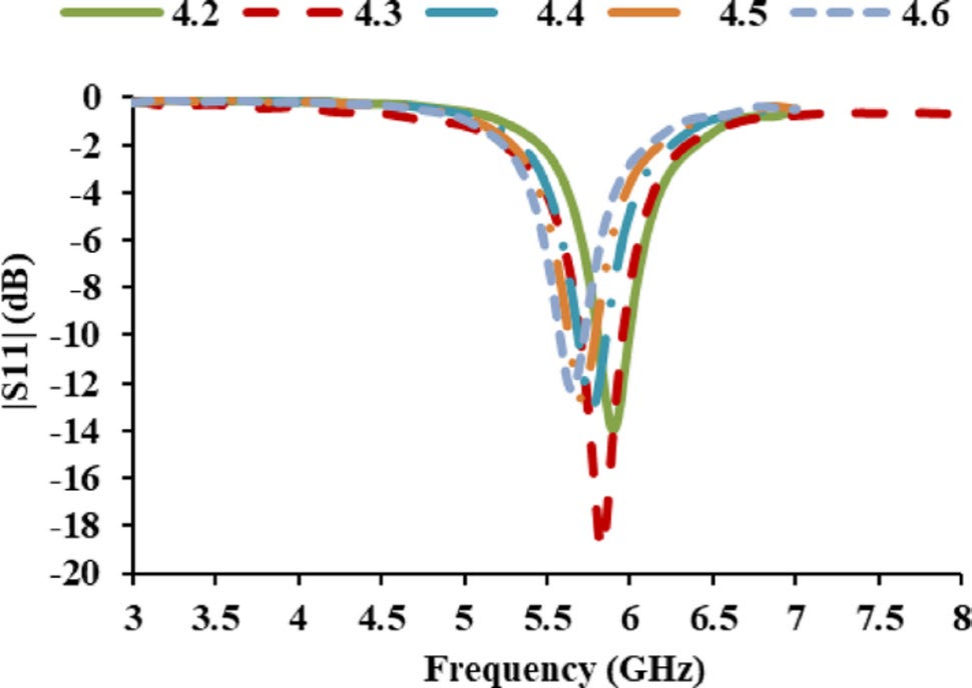
Reflection coefficient with respect to the FR-4 permittivity.

**Fig. 4:**
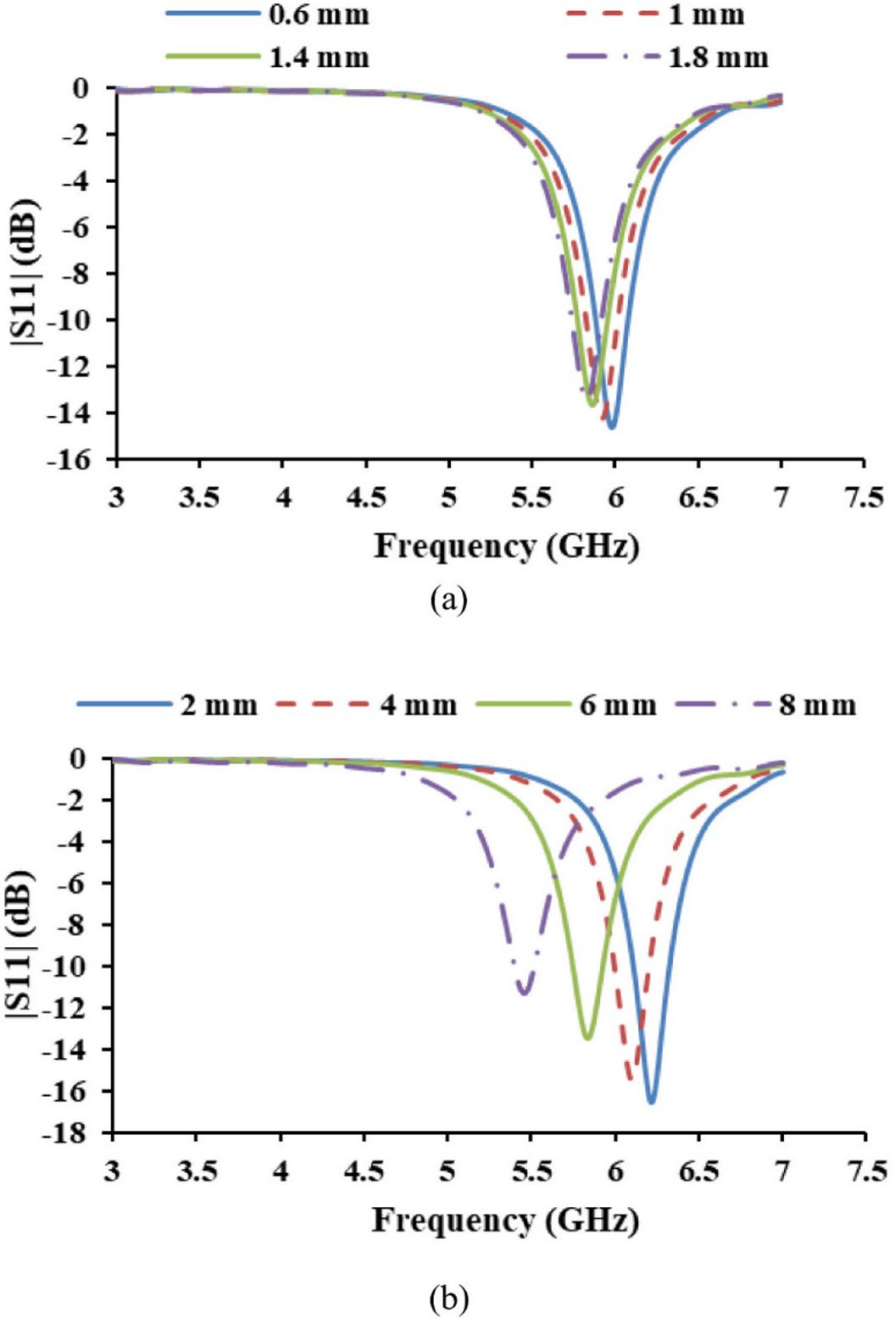
Reflection coefficient with respect change in (**a**) Slot length, (**b**) Slot width.

Figure 3 depicts the reflection coefficients in relation to the permittivity value of the substrate, which ranged from 4.2 to 4.6. The typical permittivity for FR-4 is 4.3. The results show a minor deviation from permittivity. Figure 4a and b depict the reflection coefficients as it relates to the change in slot width and length. According to the results, the slot width and length must be 1.8 mm and 6 mm, respectively, for the best reflection coefficient values.

### Development of the MIMO antenna

**Fig. 5:**
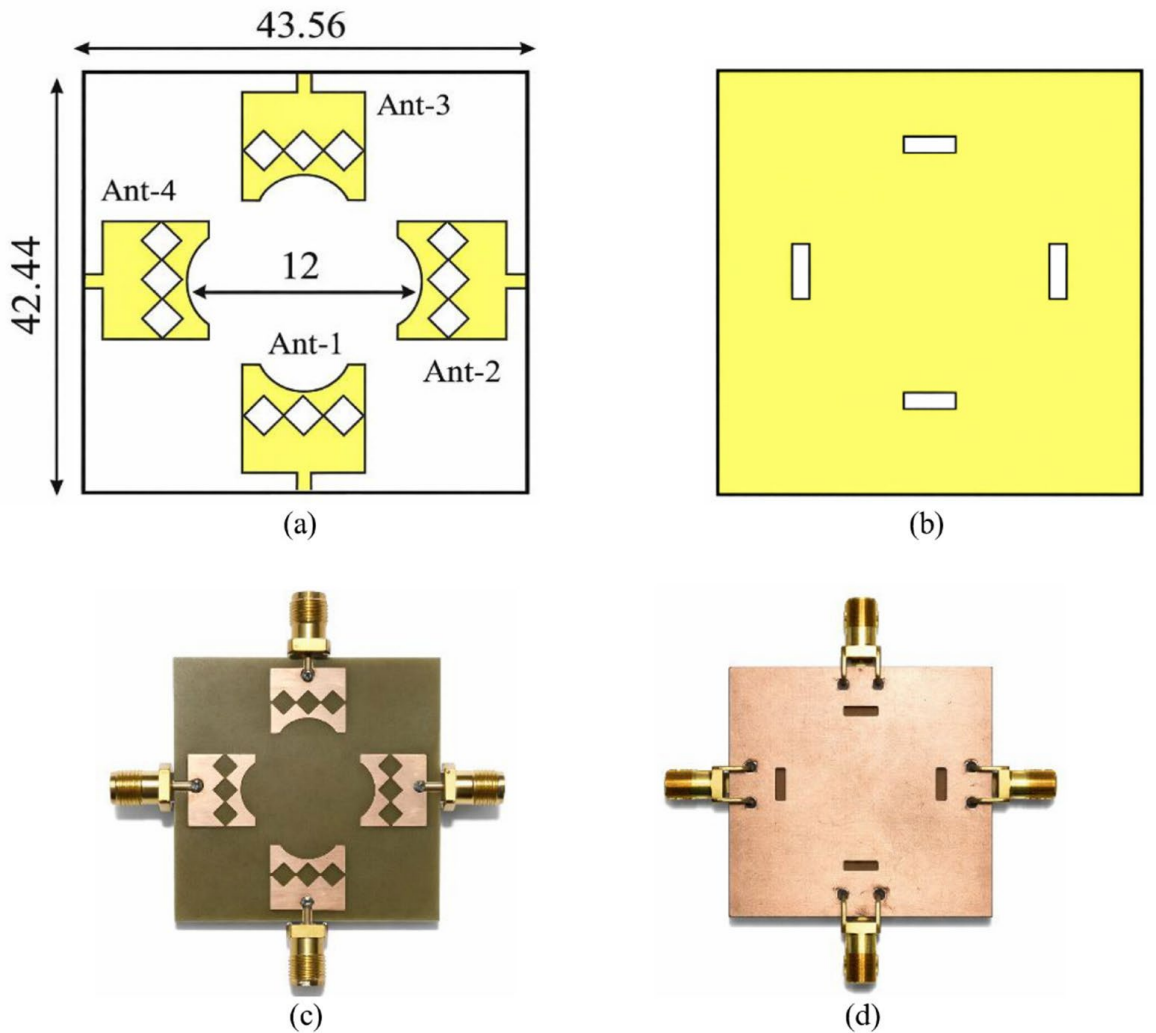
Designed tetra-port MIMO antenna: (**a**) Front view, (**b**) Back view, (**c**) Front view of the fabricated MIMO antenna, (**d**) Back view of the fabricated MIMO antenna.

Figure 5a and b depict the front and back side designs of the MIMO antenna. The proposed unit cell element is developed into a tetra-port MIMO antenna with dimensions of 43.56 mm × 42.44 mm. Each antenna is placed perpendicular to the next one, which makes the antenna highly isolated, resulting in improved mutual coupling. In the proposed MIMO antenna design, high isolation is achieved without the use of additional decoupling or isolation structures. Instead, the antenna geometry itself is carefully optimized to minimize mutual coupling between the radiating elements. The orthogonal placement of the antenna elements ensures that the surface currents are directed away from adjacent elements, which naturally reduces coupling.

The introduction of slots in the patches plays a significant role in controlling the surface current paths. These slots disturb the direct current flow toward neighbouring elements, thereby limiting energy transfer between ports. As observed from the current distribution (Fig. 2f), the currents are mainly confined around the radiator edges, with minimal interaction in the central region of the structure. Furthermore, the symmetrical arrangement of the antenna elements around the central cutout provides spatial separation, which further contributes to isolation. This design approach avoids the need for extra components such as neutralization lines or parasitic elements. Therefore, the proposed tetra-port antenna maintains a compact layout, reduced fabrication complexity, and stable radiation performance while still providing adequate isolation for MIMO operation. The interelement spacing of the MIMO antenna is 12 mm. The Keysight N9926A Vector Network Analyzer is used for reflection coefficients measurement. For ensuring the measurement accuracy, a full two port calibration is performed using the short open load through (SOLT) calibration method before measurements. Anechoic chamber of size 7 m × 3 m × 2.6 m operating in the frequency range of 800 MHz to 40 GHz is used for the radiation pattern measurements. The measurement is done using the test procedure STD-IEEE-299. The distance between the transmitter and the receiver is 10 m. In the chamber, a 1-axis fixed transmitter positioner system and a 5-axis rotary receiver positioner system are used.

**Fig. 6:**
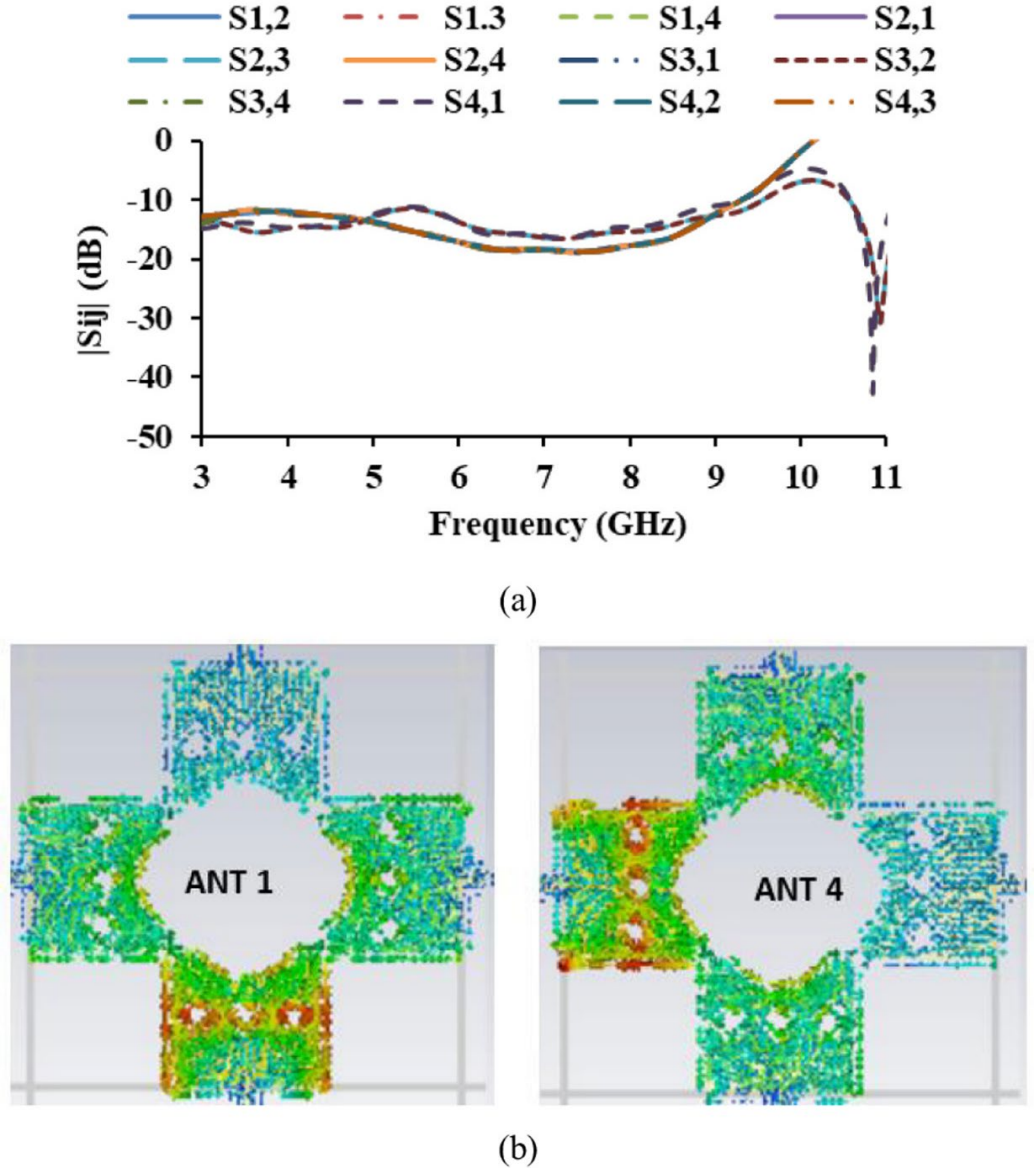
(**a**) Mutual coupling, (**b**) Surface current at interelement spacing of 10 mm.

Figure 6a shows the mutual coupling and the surface current distribution of the MIMO antenna with interelement spacing of 10 mm. The result shows that the mutual coupling is less than −10 dB for the required frequency. The surface current distribution of the MIMO antenna shows (in Fig. 6b) that the surface current of one antenna influences the other antenna due to less spacing between the elements.

**Fig. 7:**
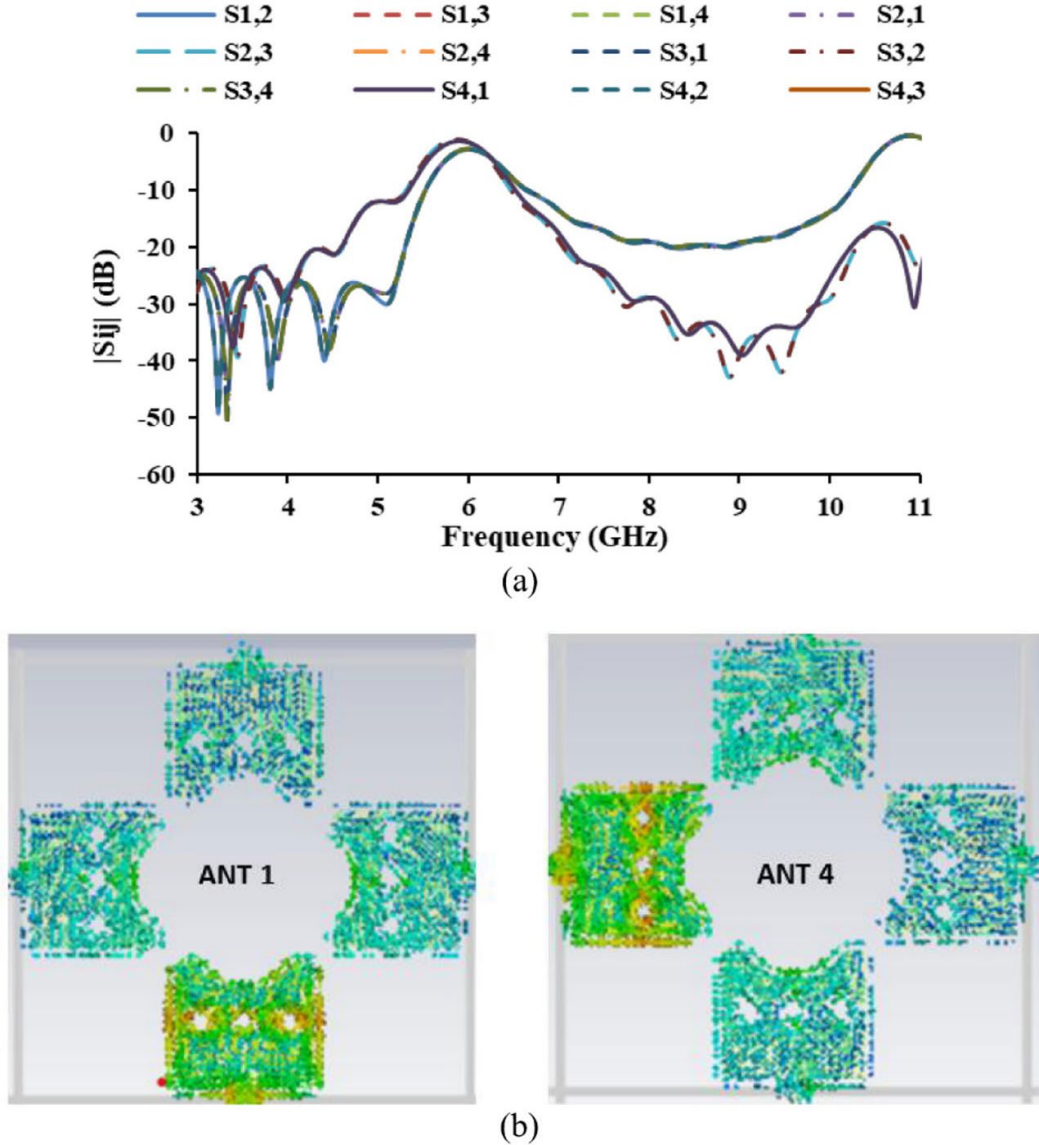
(**a**) Mutual coupling, (**b**) Surface current at interelement spacing of 11 mm.

Figure 7a depicts the mutual coupling and surface current distribution of the MIMO antenna with an interelement spacing of 11 mm. The isolation is insufficient, as the isolation is less than 15 dB. The surface current distribution of the MIMO antenna shows (in Fig. 7b) that the surface current of one antenna influences the other antenna elements.

The proposed MIMO antenna is compared to other tetra-port antennas in the literature to understand the uniqueness of the proposed antenna in terms of size and isolation structures.

**Table 1:** Comparison with tetra-port MIMO antennas reported in the literature.

Ref.	Frequency (GHz)	Ports	Isolation (dB)	Size (λ_0_^3^)	Technique
^32^	5.8	4	> 18	1.57*λ*_0_ × 1.57*λ*_0_	Parasitic decoupling
^33^	5.9	4	> 20	0.86*λ*_0_ × 0.86*λ*_0_	Neutralization line
^34^	5.8	4	> 22	3.5*λ*_0_ × 3.5*λ*_0_	EBG structure
^35^	5.9	4	> 19	0.69*λ*_0_ × 0.69*λ*_0_	Metamaterial decoupling
Prop.	5.9	4	> 20	0.84*λ*_0_ × 0.86*λ*_0_	Slot + DGS + Orthogonal placement

As highlighted in Table 1, the originality of the proposed work compared with recently reported tetra-port vehicular MIMO antennas can be summarized as follows.

- The proposed antenna design achieves isolation greater than 20 dB using only slot loading, DGS, and orthogonal antenna placement, unlike previous studies. In literature, additional structures such as metasurfaces, EBG structures, or parasitic elements are used to improve isolation.
- The antenna is designed for the 5.9 GHz ITS/V2X band, which is crucial for DSRC, V2V, and V2I communication. This enhances the suitability of the vehicular communication system.
- The proposed antenna achieves greater than 20 dB isolation and better mutual coupling suppression in a compact configuration through orthogonal placement and minimal element spacing. The antenna is smaller than other antennas reported in literature^32–34^ for the operating frequency.

## Results and discussion

### Radiation characteristics of the antenna

**Fig. 8:**
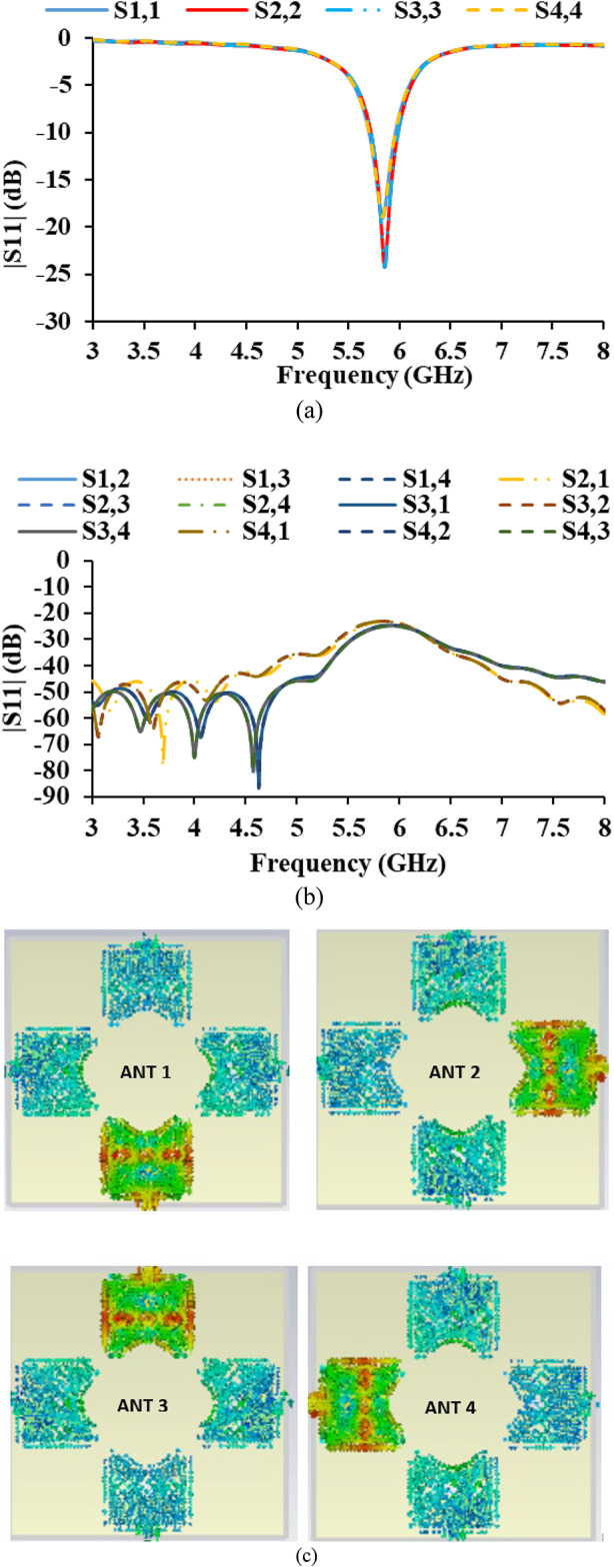
Designed tetra-port MIMO antenna: (**a**) Reflection coefficients, (**b**) Mutual coupling, (**c**) Surface current.

Figure 8 shows the measured reflection coefficients and mutual coupling parameters of the tetra-port MIMO antenna. The reflection coefficients (Fig. 8a) of all four antenna elements exhibit clear resonance at 5.9 GHz, indicating that each element operates at the intended frequency with stable and consistent performance. The close agreement in resonant behavior confirms the effectiveness of the antenna geometry and symmetry in maintaining uniform characteristics among the elements. Furthermore, the mutual coupling (Fig. 8b) between the antenna ports is well suppressed, with isolation values exceeding 20 dB at the desired frequency. This high level of isolation demonstrates that the proposed MIMO antenna design effectively minimizes interaction between elements (Fig. 8c), making it suitable for reliable multi-port vehicular communication applications.

**Fig. 9:**
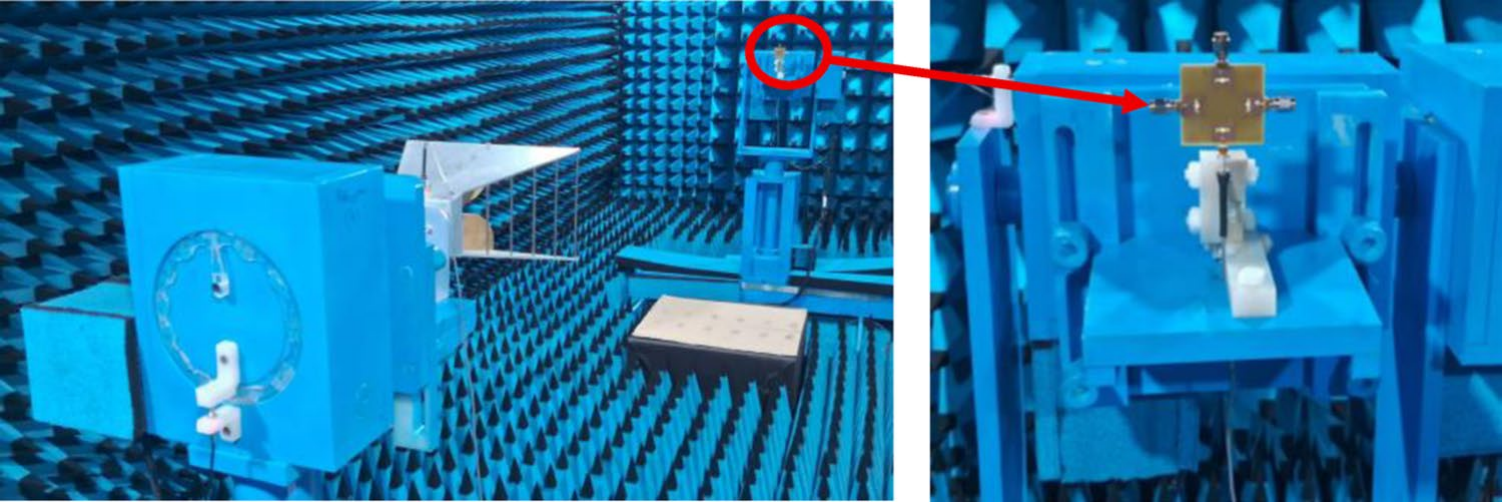
Anechoic chamber measurements of the MIMO antenna.

**Fig. 10:**
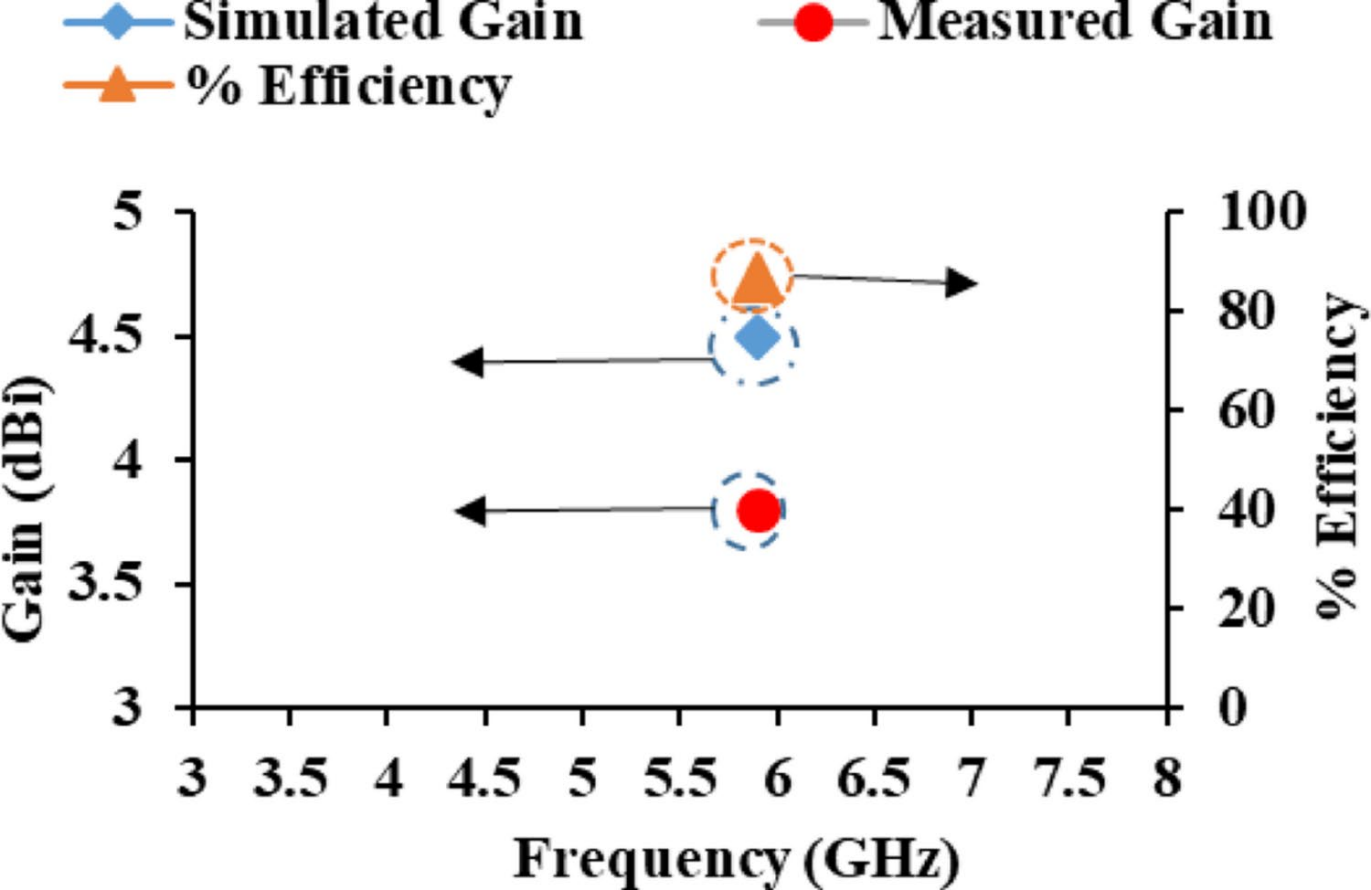
Gain and efficiency of the antenna.

The far-field measurements of the tetra-port MIMO antenna are carried out in the anechoic chamber, as shown in Fig. 9. The suggested tetra-port antenna exhibits a measured gain of 3.8 dBi and a radiation efficiency of 87% at the operating frequency. These values indicate efficient radiation performance and low power loss within the tetra-port antenna configuration. The achieved gain is suitable for vehicular communication applications, where stable signal coverage and reliable link quality are required. The high radiation efficiency confirms that most of the input power is effectively converted into radiated energy, which can be attributed to the optimized patch geometry and well-controlled current distribution. The gain and efficiency variations across the operating band are presented in Fig. 10, demonstrating consistent performance and validati ng the suitability of the antenna for practical MIMO and V2X communication systems.

**Fig. 11:**
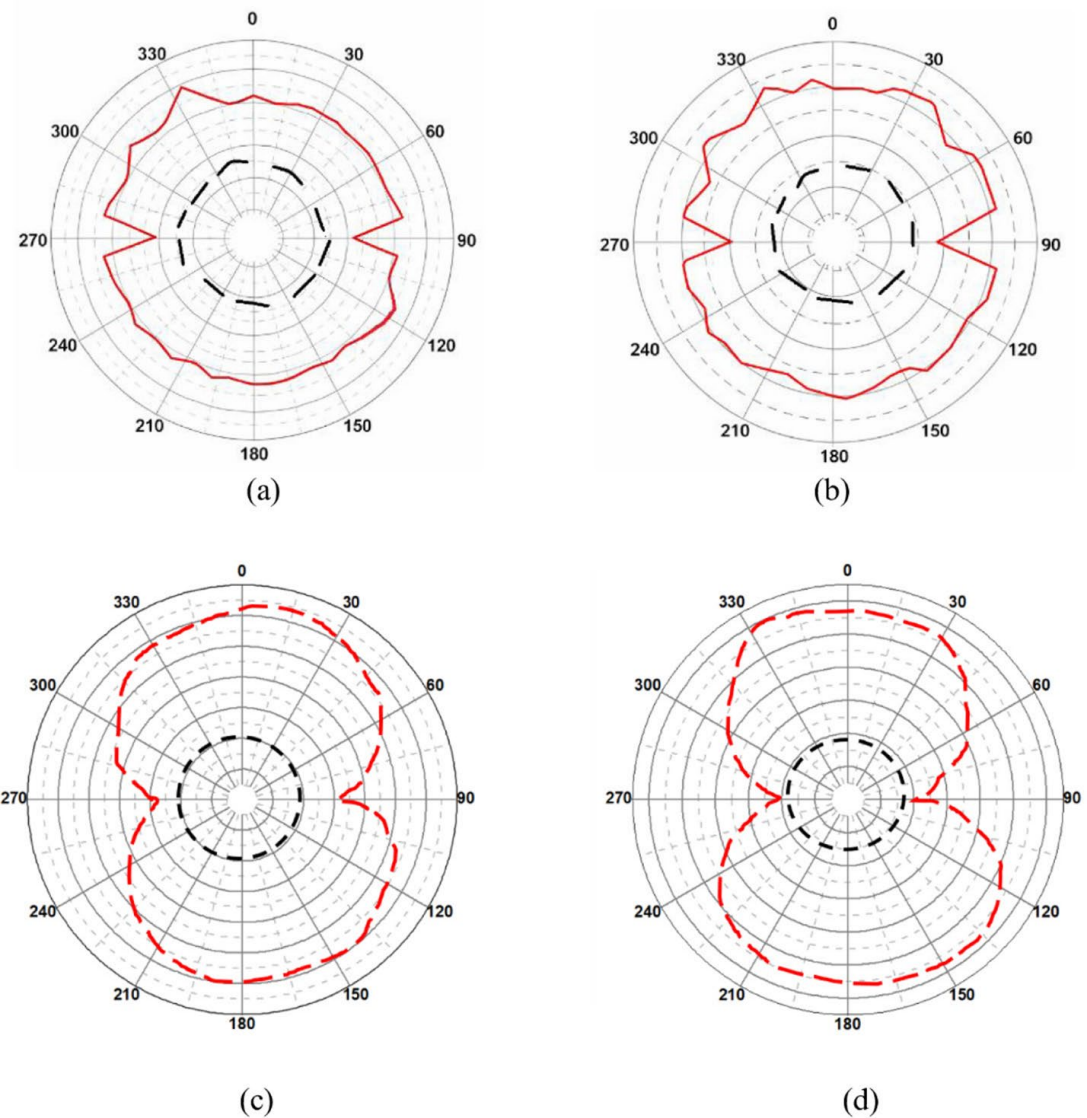
Radiation characteristics of the antenna (dB): (**a**) Measured E-plane, (**b**) Measured H-plane (**c**) Simulated E-plane, (**d**) Simulated H-plane (thick line: co-pol, split line: cross-pol).

The E-plane and H-plane radiation characteristics of the proposed antenna are presented in Fig. 11. The radiation patterns demonstrate stable and well-defined directional behavior, confirming that the antenna radiates efficiently at the intended operating frequency. The measured patterns closely follow the expected radiation characteristics of the design, indicating reliable performance.

### Diversity characteristics

To understand the performance of the MIMO antenna, it is necessary to investigate the diversity parameters. The diversity parameters of the MIMO antenna include ECC, DG, TARC, and CCL^36,37^. ECC is an important diversity parameter that indicates how two antennas are independent. The ideal value for ECC is less than 0.1. ECC is calculated using the far-field, as shown in Eq. (1).

1 $$ECC=~\frac{{{{\left| {\int {\int {\left[ {\overrightarrow {{F_1}} \left( {\theta ,\varphi } \right) \cdot \overrightarrow {{F_2}} \left( {\theta ,\varphi } \right)} \right]} } d\Omega } \right|}^2}}}{{\int {\int {{{\left| {\overrightarrow {{F_{1~}}} \left( {\theta ,\varphi } \right)} \right|}^2}d\Omega \int {\int {{{\left| {\overrightarrow {{F_2}} \left( {\theta ,\varphi } \right)} \right|}^2}d\Omega } } } } }}$$

The signal acquired by the desired antenna element is compared to the signal acquired by the combination of all other antenna elements to calculate the signal strength in the form of DG. The DG value should be greater than 9.9 dB. The DG is calculated using Eq. (2).

2 $$DG=10\sqrt {1 - {{\left| {ECC} \right|}^2}}$$

In a MIMO system, the diversity parameter metric known as TARC is used to determine the impact of one element on another. The TARC (Eq. (3)) value should be less than −10 dB across the operating frequency range.

3 $$TARC=~\frac{{\sqrt {\mathop \sum \nolimits_{{i=1}}^{N} {{\left| {{b_i}} \right|}^2}} }}{{\sqrt {\mathop \sum \nolimits_{{i=1}}^{N} {{\left| {{a_i}} \right|}^2}} }}$$

The correlation in MIMO antenna configuration will result in capacity loss. To understand capacity loss, the metric known as CCL is investigated. It is calculated using Eq. (4), and the value should be less than 0.4 bits/s/Hz.

4 $$CCL=~ - lo{g_2}\left| {{\Psi ^R}} \right|$$

**Fig. 12:**
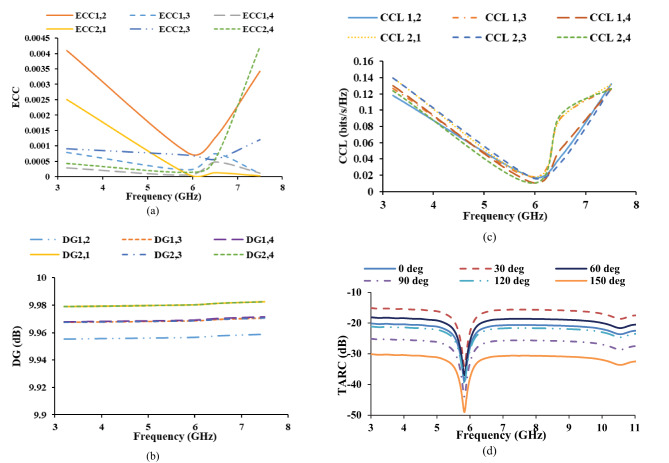
Simulated Diversity parameters of the tetra-port MIMO antenna: (**a**) ECC, (**b**) DG, (**c**) CCL, (**d**) TARC.

**Fig. 13:**
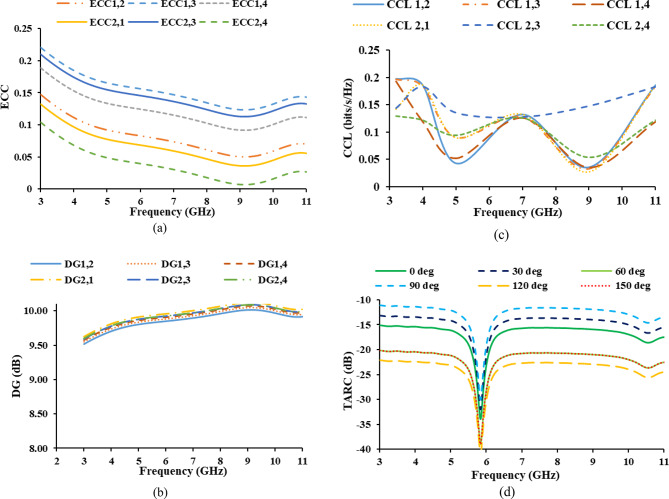
Measured Diversity parameters of the tetra-port MIMO antenna: (**a**) ECC, (**b**) DG, (**c**) CCL, (**d**) TARC.

Figures 12 and 13 depicts the diversity parameter plots. The results are plotted with respect to antennas 1 and 2. Antenna 3 and antenna 4 have the same orientation and characteristics as antennas 1 and 2, respectively.

The mean effective gain (MEG) measures the ability of the antenna to receive transmitted electromagnetic power. The MEG is calculated using Eq. (5), and the value of the ratio should be less than 1. The MEG of the antenna is given in Tables 2 and 3.

5 $$MEG=\mathop \smallint \limits_{0}^{{2\pi }} \mathop \smallint \limits_{0}^{\pi } \left[ {\frac{{XPR}}{{1+XPR}}{G_\theta }\left( {\theta ,\Phi } \right){P_\theta }\left( {\theta ,\Phi } \right)+\frac{1}{{1+XPR}}{G_\emptyset }\left( {\theta ,\Phi } \right){P_\emptyset }\left( {\theta ,\Phi } \right)} \right]\sin \theta d\theta d\Phi$$

**Table 2:** Simulated MEG of the MIMO antenna relating to antenna 1 and antenna 2.

MEG of Antenna	Isotropic XPR = 0 dB MEG1/MEG2	Outdoor XPR = 1 dB MEG1/MEG2	Indoor XPR = 5 dB MEG1/MEG2
MEG1/MEG2	1	0.999	1
MEG1/MEG3	1	1	1
MEG1/MEG4	1	1	0.999
MEG2/MEG1	0.999	0.999	1
MEG2/MEG3	1	1	0.999
MEG2/MEG4	1	1	1

**Table 3:** Measured MEG of the MIMO antenna relating to antenna 1 and antenna 2.

MEG of Antenna	Isotropic XPR = 0 dB MEG1/MEG2	Outdoor XPR = 1 dB MEG1/MEG2	Indoor XPR = 5 dB MEG1/MEG2
MEG1/MEG2	1	0.998	1
MEG1/MEG3	0.998	1	0.997
MEG1/MEG4	1	0.998	1
MEG2/MEG1	0.999	0.998	1
MEG2/MEG3	0.997	1	0.997
MEG2/MEG4	1	0.994	1

The vehicular antennas are designed to operate in a highly dynamic environment where channel conditions change rapidly due to vehicle movement and surrounding infrastructure. The antenna can be subjected to the Doppler effect caused by the relative motion of the transmitter and receiver. This causes frequency shifts, which affect signal reception. Also, reflections from nearby buildings, trees, and other structures cause multipath propagation, resulting in signal fading. In dense urban canyon scenarios, additional attenuation and scattering are caused by closely spaced buildings. To provide reliable communication, the proposed vehicular antenna must maintain stable impedance and radiation characteristics. Parameters such as the reflection coefficient, gain, and radiation pattern are investigated to better understand the stability of the suggested antenna. The antenna has dual polarization, which is useful for multipath propagation. Additionally, the performance of the antenna when mounted in the vehicle is investigated.

## On-vehicle analysis of the antenna

**Fig. 14:**
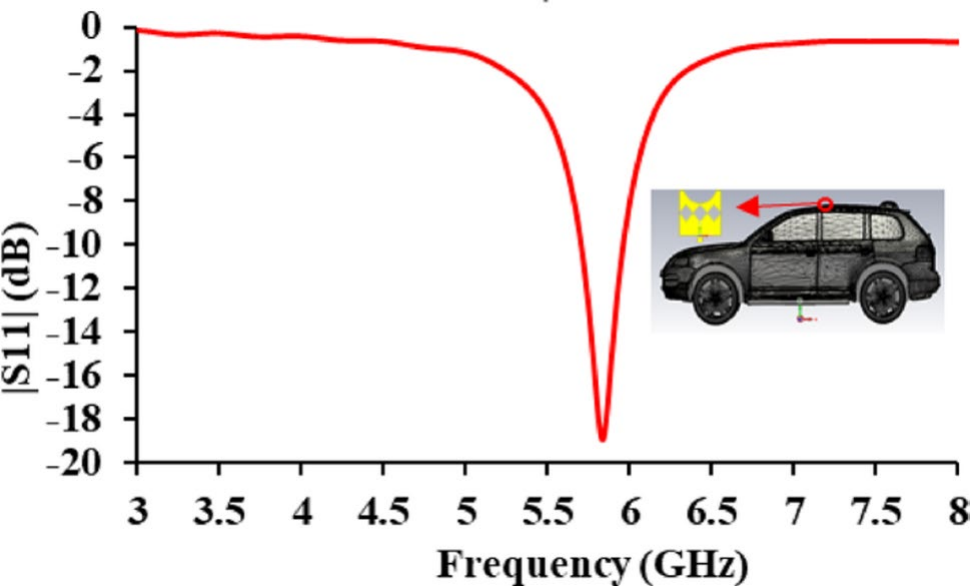
Reflection coefficients in relation to antenna placement on the windshield.

**Fig. 15:**
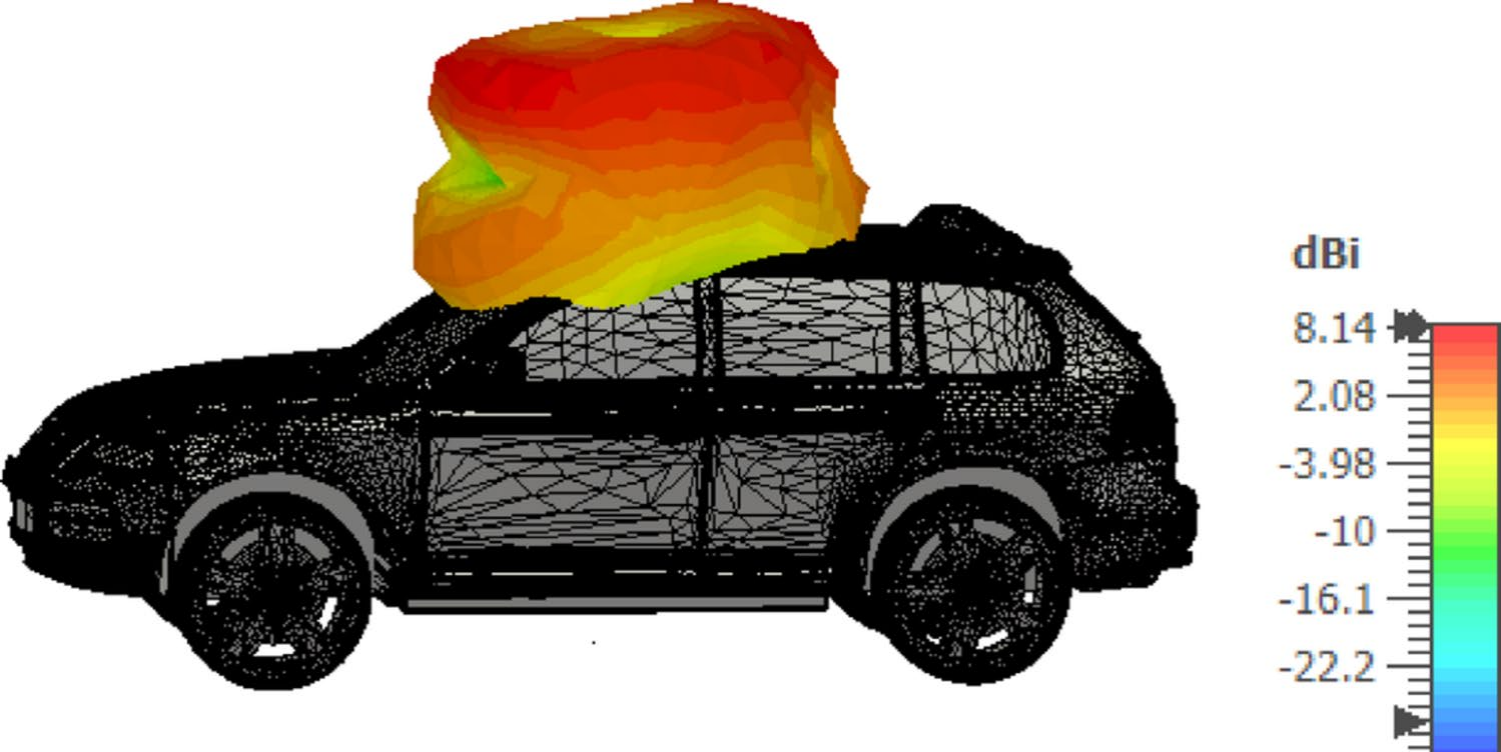
Directivity of the suggested antenna.

The on-car performance of the suggested antenna is investigated by placing it on the roof of a 3D car model. The antenna can be placed on the roof, bumper, side mirror, or windshield of the vehicle. To avoid ground loss, the best solution is to place the antenna on the roof. The roof of the vehicle acts as a large ground plane, affecting the properties of the antenna. An open source 3D model is used to mimic the vehicle. The metal body of the car model is given as perfect electric conductor (PEC). The designed antenna is placed on the roof of the 3D car model, and the reflection coefficients and directivity are studied. The results show that the performance of the suggested antenna remains stable even after it is mounted on the vehicle. Figure 14 depicts the reflection coefficients of the antenna with respect to vehicle analysis. The directivity of the suggested antenna for on-vehicle analysis is investigated (Fig. 15), and the results show that the antenna exhibits omnidirectional characteristics.

**Fig. 16:**
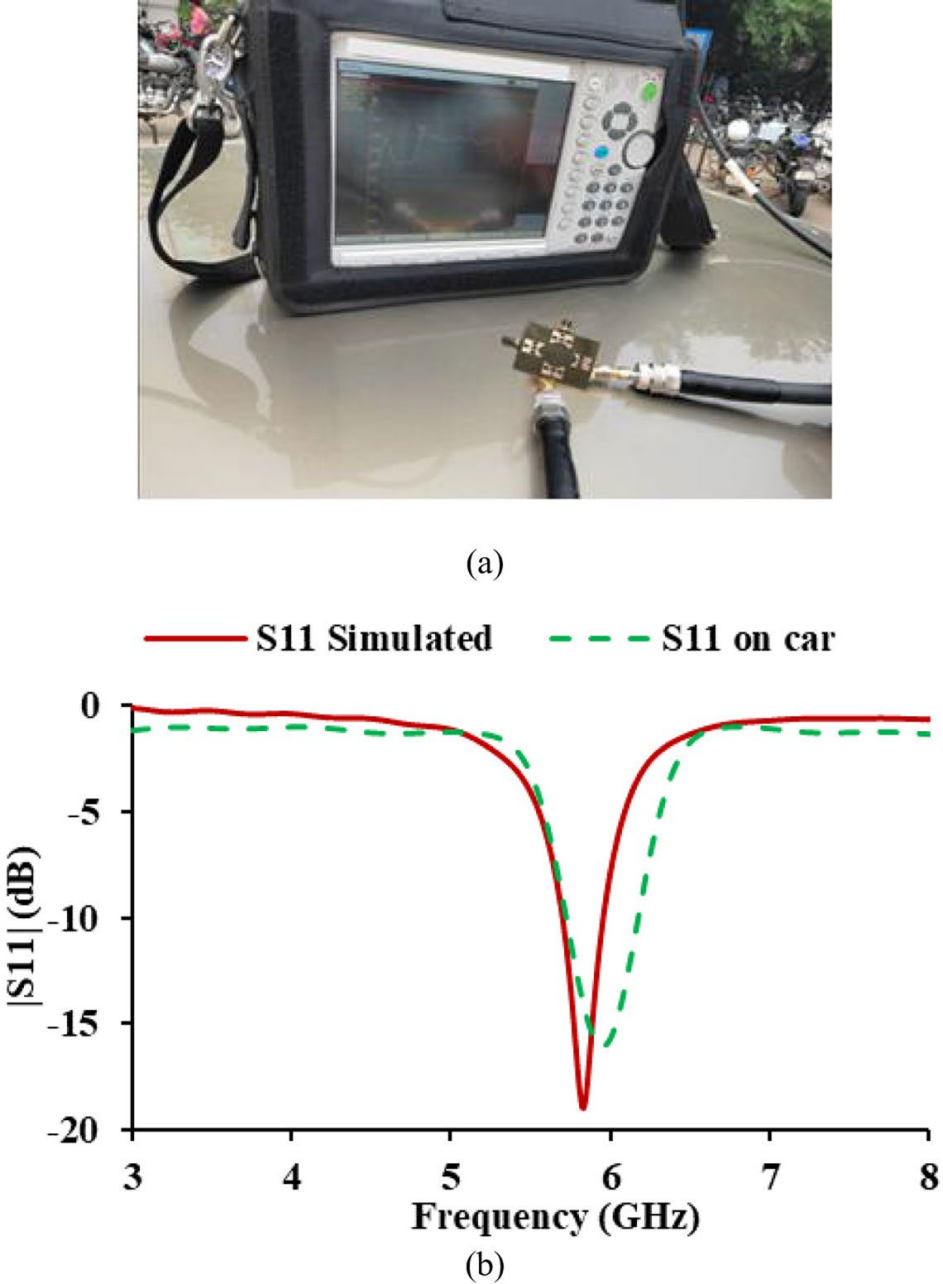
(**a**) On-car measurement setup, (**b**) Reflection coefficients.

The reflection coefficients of the antenna measured in real time on the car show a slight deviation due to environmental factors. However, the required bandwidth has been achieved. Figure 16a shows the on-car measurements, while Fig. 16b shows the corresponding reflection coefficients of the suggested antenna.

Table 4 presents the salient features and performance parameters of the proposed tetra-port MIMO antenna.

- The suggested tetra-port MIMO antenna measures 43.56 mm × 42.44 mm, resulting in a smaller area compared to previous studies^23,38,41,44-48^. Unlike most reported designs that rely on expensive substrates to achieve high isolation and gain, the proposed antenna attains comparable or superior performance using low-cost FR-4, making it highly attractive for practical and large-scale wireless deployments.
- While several reported antennas operate over ultra-wide or multiband ranges, the suggested tetra-port antenna design focuses on 5.9 GHz, resulting in superior isolation, correlation, and efficiency metrics, making it ideal for targeted applications such as ITS, WLAN, or 5G sub-6 GHz systems.
- The proposed antenna achieves ECC < 0.0082 and DG > 9.9 dB with four elements, outperforming or matching prior works that either use fewer elements or larger apertures. This highlights the effectiveness of the isolation and decoupling mechanism without increasing system complexity.
- Many related works focus on two-element MIMO systems^22,38,39,42,43^. The suggested tetra-port antenna demonstrates that high channel capacity and low mutual coupling can be preserved even in a four-element configuration, which is more relevant for modern high-data-rate wireless systems.
- The antenna provides 87% radiation efficiency and 3.8 dBi gain while maintaining a moderate and application-friendly size. Compared to prior compact designs that sacrifice efficiency or isolation, this work achieves a well-optimized balance across all key metrics.
- With a TARC below −13.5 dB and CCL < 0.28 bits/s/Hz, the proposed design demonstrates excellent MIMO signal integrity and minimal correlation losses, surpassing many existing FR-4 based designs and even several RT5880-based antennas.
- The combination of low ECC, high efficiency, compact size, and FR-4 substrate place the suggested antenna as a practically deployable solution, rather than a purely experimental design requiring specialized materials. The proposed antenna is a reliable solution for 5G vehicular communications and a foundation for future 6G vehicular applications.

**Table 4:** Comparison of the proposed antenna to existing literature.

Refs.	Size (mm × mm)	Substrate	Frequency (GHz)	Gain (dBi)	Efficiency (%)	No. of Elements	ECC	DG (dB)	TARC (dB)	CCL (bits/s/Hz)
^23^	60 × 28	Rogers 5880	26−40	> 4	> 80	4	< 0.021	> 9.9	< −13	< 0.4
^38^	28 × 60	FR-4	3.7−7.6	4.6	> 80	2	< 0.051	–	–	< 0.4
^39^	6 × 17.37	Rogers 5880	33.8−39.6	> 5	> 90	2	< 0.031	> 9.9	< −10	< 0.35
^16^	28 × 28	FR-4	3.5/5.2	3.5/4	88/91	4	< 0.0001	> 8	–	–
^40^	27 × 38.64	Rogers 5880	3.7−11	6.5	> 60	2	< 0.1	> 9	–	< 0.3
^22^	9.2 × 18	Rogers 5880	24.8−28.6, 36.2−40.8	> 6	–	2	< 0.0001	> 9	–	< 0.4
^41^	60 × 40	FR-4	2.7−4, 6.9−7.5	4	80	2	< 0.02	> 9.9	<−10	< 0.4
^42^	32 × 26	FR-4	2.8−14	6.2	82	2	< 0.0012	> 9.9	< −10	< 0.4
^43^	58 × 28	FR-4	3.24−14.39	6.1	> 87	2	< 0.05	> 9	< −10	< 0.4
^44^	55 × 30	FR-4	2.8−4.5	2.5	90	4	< 0.005	> 9.9	< −10	< 0.4
^45^	56 × 56	FR-4	2.96−13.2	3	90	4	< 0.005	> 9.9	< −10	< 0.4
^46^	45 × 48	FR-4	5.8−6.2	5	> 82	4	< 0.03	> 9.7	< −11	< 0.35
^47^	70 × 70	Rogers 4003	5.7−6.3	7	> 88	4	< 0.02	> 9.9	< −12	< 0.3
^48^	42 × 60	Rogers 5880	3.4−6.8	5	> 85	4	< 0.025	> 9.8	< −10	< 0.35
Prop.	43.56 × 42.44	FR-4	5.9	3.8	87	4	< 0.0045	> 9.9	< −13.5	< 0.28

## Conclusion

The proposed quad-port 5G/6G MIMO antenna operates at a frequency of 5.9 GHz. In the suggested antenna, rhombus-shaped slots are used in the radiator to improve impedance matching. A semicircular cut is made in the radiator to achieve the desired operating frequency. The unit cell antenna has dimensions of 12 mm × 11 mm. The proposed antenna has measured gain and efficiency of 3.8 dBi and 87%, respectively. The tetra-port MIMO antenna measures 43.56 mm × 42.44 mm, with an interelement spacing of 12 mm. The antenna provides good isolation (> 20 dB) without the need for any additional structures. The diversity parameters of the MIMO antenna are investigated, and the findings indicate that the antenna is suitable for vehicular communication. The proposed MIMO antenna exhibits an envelope correlation coefficient below 0.1, a diversity gain above 9.9 dB, a total active reflection coefficient below −10 dB, channel capacity loss below 0.4 bits/s/Hz and MEG ratio is close to unity. The on-board analysis of the antenna is investigated to better understand its performance after it has been installed in the vehicle. The results demonstrate that the antenna is appropriate for automotive applications.

## Data Availability

All the data are withing the manuscript itself.
